# A Cross-sectional Study on the Effectiveness of Two Different Tooth-brushing Exercise Methods in Blind Childrendren

**DOI:** 10.1055/s-0043-1768469

**Published:** 2023-06-13

**Authors:** Kurniaty Pamewa, Iwan Ahmad Musnamirwan, Arlette Suzy Setiawan

**Affiliations:** 1 Pediatric Dentistry Residency Program, Faculty of Dentistry Universitas Padjadjaran, Jl. Sekeloa Selatan 1, Bandung, Indonesia; 2 Department of Pediatric Dentistry, Faculty of Dentistry, Universitas Muslim Indonesia, Yayasan Wakaf, Jl. Pajonga Dg. Ngalle No.27, Pa'batong, Kec. Mamajang, Kota Makassar, Sulawesi Selatan, Indonesia; 3 Department of Pediatric Dentistry, Faculty of Dentistry, Universitas Padjadjaran, Jl. Sekeloa Selatan 1, Bandung, Indonesia

**Keywords:** tooth-brushing, health behavior, oral hygiene, blindness

## Abstract

**Objective** Blind children tend to have poor oral health. Oral health education is needed to reduce the prevalence of dental caries and periodontal diseases among blind children. The aim of this study was to evaluate the effectiveness of two tooth brushing exercise methods toward blind children’s knowledge, attitude, behavior, and oral hygiene.

**Materials and Methods** The purposive sampling technique was used in this study on 80 blind children aged between 7 and 16. Children were divided into two groups of 40 children each. In group I, children received the tooth-brushing exercise through the Braille–verbal method, and group II received the tactile–verbal method. Their knowledge, behavior, and attitude were recorded by a questionnaire, and their oral hygiene was assessed during a personal oral examination. Data were analyzed using Wilcoxon–Mann–Whitney non-parametric test.

**Results** Differences in effectiveness toward knowledge, attitude, and oral hygiene were found between both methods with the following values: *p* -value = 0.04 (<0.05), 0.04 (<0.05), and 0.0002 (<0.05). No difference in effectiveness toward behavior was found: *p* -value 0.30 (>0.05).

**Conclusion** The two tooth-brushing methods could change knowledge, attitude, and oral hygiene in blind children. The tactile–verbal method was more effective than the Braille–verbal method in changing blind children's oral hygiene.

## Introduction

Blindness in children can cause various problems related to their oral health. Blind children generally have poorer oral health compared to nonblind children. 1 Poor oral health in blind children can be caused by lack of parental guidance during their children's dental cleaning activity, lack of children's motor skills, lack of awareness of the need for a regular dental checkup, lack of knowledge about oral health, and improper dental cleaning technique. 2 3 Oral health education, which includes tooth-brushing exercise, is needed as a preventive measure to reduce the prevalence of dental caries and periodontal diseases in blind children. 4 5 Oral health education aims to promote the knowledge and attitude required to develop a behavioral pattern that can better ensure oral health. 6 7

Blind children generally have problems understanding and mastering oral hygiene practice techniques. Therefore, several studies have been devoted to exploring pediatric oral health intervention methods. Based on these studies, the most appropriate oral health education methods for blind children are the verbal method, Braille method, and tactile demonstration. While the verbal, oral health education method involves giving spoken instructions, the Braille method uses the Braille alphabet system to teach blind children to maintain their oral hygiene. The tactile method requires tactile demonstration using a toothed doll as a medium. 8 9 10 11 12 It is assumed that if the tooth-brushing exercise method is delivered in a combined form of Braille and verbal or tactile and verbal, it will lead to a different result. Thus, this study was conducted to evaluate the difference in effectiveness between the Braille–verbal and tactile–verbal tooth-brushing exercise methods toward knowledge, attitude, behavior, and oral hygiene in a group of blind children.

## Materials and Methods

### Participant Recruitment

Prior informed written consent was obtained from school authorities and the children's parents. A total of 80 (7–16-year-old) children were selected out of 86 institutionalized children, using a purposive sampling method from a Special School for Blind in Bandung, Indonesia. Children who are blind since birth (legally blind) and free from any other form of mental or physical handicapping conditions were included in the study. Children with visual impairment associated with any systemic condition or any other disability were excluded from the study. Since the study involved dental debris and calculus measurement, children with dental caries on the particular teeth to be examined were also excluded. Participation in the study was voluntary.

### Instruments Development

A set of structured questions were asked to each child to assess their knowledge, attitude, and behavior on the importance of oral hygiene and personal experience with dental care. The questionnaire consists of 15 item knowledge questions and 10 items each for attitude and behavior questions. Items for knowledge and behavior were scored with the Guttman scale (scored 0 for the wrong answer, 1 for the correct answer), whereas attitude items were scored by 5 Likert-scale. The maximum score for knowledge was 15 with a 0 to 5 classified as standard knowledge, 6 to 10 moderate knowledge, and 11 to 15 high knowledge. While the maximum score for behavior was 10, and the higher score indicates better oral health behavior. These also applied to attitude; the higher value of attitude response indicates a better attitude toward oral health. All items have been validated and pre-tested with Cronbach alpha of 0.87.

The responses were recorded through one-to-one personal interviews. Greene-Vermillion Simplified Oral Hygiene Index was applied to each child in the oral examination by using disclosing solution to the tooth surface after the interview to assess their oral hygiene level. The children were then divided into two groups of 40 children each by purposive sampling method.

Group I: Children received tooth-brushing instruction through a Braille written book to be read aloud by each child and followed by the investigator's verbal explanation.

**Fig. 1:**
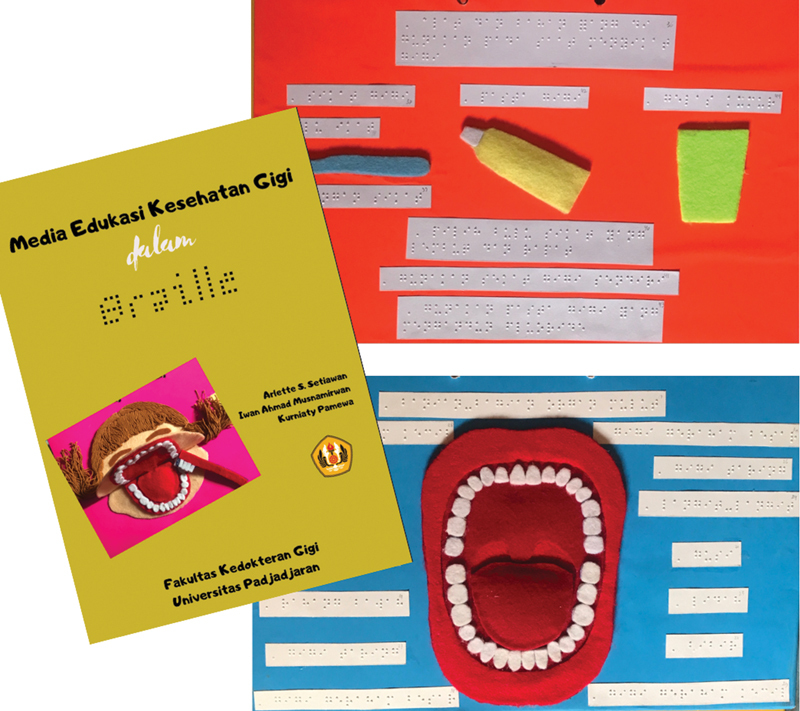
Braille book.

The book was composed by the investigators, and it includes information on oral hygiene maintenance and exercise (including Fones tooth brushing technique) written in Braille text and tactile picture ( Fig. 1 ). Braille text was made at the Indonesian Braille Publishing Center. Tactile pictures in the book were designed by the investigators using flannel. The book was first tested on four subjects before study.

**Fig. 2:**
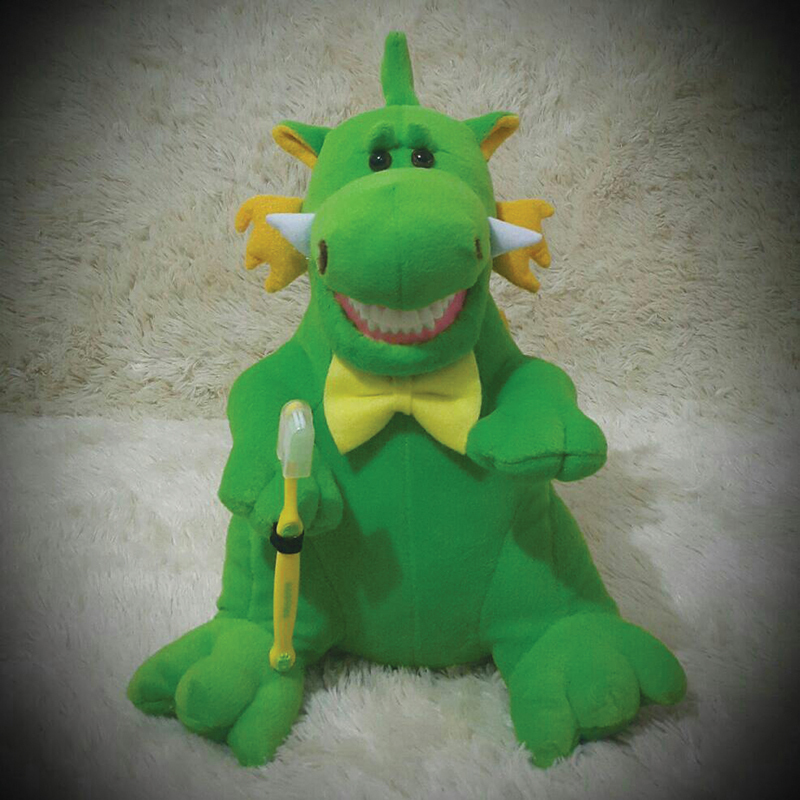
Tooth model doll.

Group II: Children received tooth brushing instruction using a tooth model doll ( Fig. 2 ) and verbal instructions from the investigator. The demonstration includes Fones circular motion technique of tooth brushing by an investigator on a tooth model doll. Each child held the investigator's hand with their working hand while placing the other hand on the doll's teeth. Each participant (both in group I and II) was asked to perform the tooth-brushing method they learned with the investigator's guidance.

The tooth brushing exercise was imparted twice weekly in three consecutive weeks. This study was conducted by two investigators; one investigator provides verbal instruction and guidance. The second investigator, unaware of how the child was educated, recorded the participant's responses on a questionnaire and oral examination at the beginning and end of the study. Data obtained from the questionnaire was subjected to statistical analysis by the Wilcoxon–Mann–Whitney non-parametric test. Concerning oral hygiene, data was analyzed by unpaired t-test.

## Results

Children's responses to the questionnaire and oral examination are shown in Table 1 . In both groups, children show improved knowledge, attitude, and behavior after being introduced to the given tooth-brushing exercise method. Their oral hygiene index decreased after exposure to both tooth-brushing exercise methods .

**Table 1:** Children's knowledge, attitude, behavior, and oral hygiene before and after exposure to two tooth-brushing exercise methods

	Group I Braille–verbal		Group II tactile–verbal		
	Before	After	Before	After
**Knowledge** **SD** n	8.2 1.75 20	14.6 0.96 20	8 2.11 20	10.8 2.57 20
**Attitude**				
**SD** ***n***	34.6 6.06 20	41.6 5.38 20	34.9 5.99 20	39.2 5.96 20
**Behavior**				
**SD** ***n***	4.9 0.57 20	5.3 0.32 20	4.7 0.67 20	5.1 0.71 20
**Oral hygiene**				
**SD** ***n***	2.8 1.03 20	1.8 0.60 20	2.9 0.70 20	1.3 1.04 20

Abbreviation: SD, standard deviation.

Table 2 show a significant difference in effectiveness toward knowledge ( *p* -value = 0.04) and attitude ( *p* -value = 0.04), as for behavior ( *p* -value = 0.3) was insignificant. An unpaired *t* -test was then used to measure group I and group II's difference in their effectiveness toward oral hygiene. Based on the test results, there was a statistical significance difference (p-value = 0.0002) between both groups.

**Table 2:** Analysis of the difference in effectiveness toward knowledge, attitude, behavior, and oral hygiene between tactile and verbal tooth-brushing exercise methods

Group	Knowledge	Attitude	Behavior	Oral hygiene
**Group I**	13.1	15.2	8.7	0.65 ± 0.35
**Group II**	7.9	8.6	6.3	1.57 ± 0.52
**Group I vs. group II**	z = 2.01 p = 0.04 Significant	z = 3.57 *p* = 0.04 Significant	z = 0.93 *p* = 0.3 Insignificant	t = − 4.61 *p* = 0.0002 Significant

Abbreviation: SD, standard deviation.

Note: Group I = Braille and verbal.

Group II = Tactile and verbal.

z = z-count value.

t = t-count value.

*p* = Confidence value (<0.05).

## Discussion

Education, in general, is an essential factor responsible for behavioral change in children. 13 Key to dental and oral disease prevention, oral hygiene education more effective for school-age children because school provides the best environment to teach them about oral hygiene. Education and promotion of dental and oral health aim to change an individual's oral hygiene behavior. 14

Brushing teeth properly and regularly is a main behavioral component that is closely associated with good oral health. Unfortunately, most blind children cannot brush their teeth properly, which results from plaque accumulation, dental caries formation, and periodontal diseases. Tooth-brushing education for blind children requires time, a unique approach, and patience. 7 Exercise in this study was given individually to each child subject. Motoric skills education for blind children would be more effective if given on an individual basis. 15

The study adopted Fones tooth-brushing method because it is more understandable and applicable for people with lower motoric skills, including blind children. Joybell et al maintain that Fones and modified Bass methods prove very useful in improving blind children's dental and oral hygiene. Studies on various media, including verbal instructions, audio aids, and tactile aids, in blind children's education. Education media refer to aids used to help convey health messages, achieve better goals, and reduce obstacles to understanding messages. 16

This study used direct and indirect tactile educational media, a Braille book, and a toothed doll to teach blind children to brush their teeth properly. The Braille book used contained information about parts of the mouth, the number of deciduous and permanent teeth, tooth-brushing tool and dentifrice, proper tooth-brushing technique, and ways to maintain dental and oral health. The study was conducted in 4 weeks, 3 consecutive weeks for the education process, and the 4th week was used to follow up the process. As Foster suggests, behavioral change can be evaluated after 21 days. 17

In terms of the difference in effectiveness between Braille–verbal and tactile–verbal tooth-brushing exercise methods, this study's conclusion was consistent with that of a study by Sabilillah et al. 18 who concluded that Dental Braille Education could change blind children's knowledge and attitude. This finding, it is suggested, could be related to Braille text and reading aloud technique in educating blind students. 19 Reading aloud technique can improve students' observation skills, vocabulary, reading comprehension, memory, and reading interest. 20 The subjects following the tactile and verbal tooth-brushing methods learned to remember the tooth-brushing technique taught through individual training.

Based on this study's results, the subjects' attitude undergoing Braille and verbal tooth-brushing exercise method generally improved better than that of the subjects given tactile–verbal method. Braille–verbal tooth-brushing method was descriptively more effective to change behavior, although no significant difference resulted from both methods' statistical analysis. The study results might have been related to the subjects' lack of awareness regarding the risks of dental and oral diseases. As Nisbet and Gick maintain, “in order for behavior to change, people must feel personally vulnerable to a health threat, view the possible consequences as severe, and see that taking action is likely to either prevent or reduce the risk.” 20 Another factor that affected the study results was the environmental factor, especially parents. Parents' lack of discipline and motivation could cause some children to maintain poor behavior.

The difference in effectiveness toward dental and oral hygiene between Braille–verbal and tactile–verbal tooth-brushing education methods in this study were consistent with Ganapathi et al, who concluded that the typically tactile–verbal tooth-brushing education method resulted in better oral hygiene than the Braille–verbal method. The finding was attributed to the fact that the tactile–verbal method involved tactile tooth-brushing demonstration and practice using a toothed-doll, improving the subjects' motoric skills. 12 Coupled with corrective feedback, the combination of spoken instructions and demonstration (physical guidance and tactile demonstration) can improve blind children's motoric skills. As Downing and Chen argue, tactile demonstration and physical guidance are modeling techniques that can help blind children develop their motoric skills. Tactile demonstration helps children feel and explore the instructor's body movements on a model or object, helping them learn and understand the skill being demonstrated. The tactile demonstration allows subjects to feel and understand the instructor's rotating hand movement, the movement's rhythm, and the hand movement's direction and precision. Physical guidance can help direct subjects' body parts to the correct position required to perform a skill accurately. 21

This study had some limitations. First of all, the subjects' age range was too broad, which could be related to differences in the subjects' cognitive and psychological levels. Second, the short follow-up time did not allow retention of knowledge, attitude, behavior, and dental and oral hygiene to be evaluated after tooth-brushing education had been completed. However, this finding is entirely meaningful as the basis for further well-structured comparative study with bigger sample size. It is hoped that more representative results will be obtained to be used as protocols in oral health education in every particular school for blinds in the country.

In conclusion, this preliminary study shows that the two tooth-brushing exercise methods could change knowledge, attitude, behavior, and dental and oral hygiene in blind children. Braille–verbal tooth-brushing exercise method was more effective than the tactile–verbal method in changing subjects' knowledge, attitude, and behavior. The tactile–verbal method was proven to be more effective in changing subjects' oral hygiene.

## Why This Paper Is Essential to Pediatric Dentists

- It shows which combine tooth-brushing method can be effective in blind children
- It highlights the need for specific tools that must be provided in the pediatric dental office, especially for the pediatric dentist who deals with blind children
- This preliminary study can help another pediatric dentist around the world who is planning to do further research based on this preliminary result

**Appendix Table A1:** Questionnaire

Dimension	Item
Knowledge	1. Healthy teeth are: a) White teeth b) Teeth that are not cavities c) Yellowish teeth 2. The right time to brush your teeth in the morning: a) After getting up in the morning b) Take a shower every morning c) After breakfast 3. How many times do we have to brush our teeth in a day: a) 1 time a day b) 2 times a day c) 3 times a day 4. The right time to brush your teeth at night: a) Every afternoon bath b) Before going to bed at night c) After dinner 5. How long does it take you to brush your teeth? a) Don't know b) About 2 minutes c) About 30 seconds 6. According to you, a good action is: a) Often eat sweet foods b) Using a toothbrush alternately. c) Brush your teeth with toothpaste containing fluoride 7. Which toothbrush is good for brushing teeth? a) The bristles are soft and dense b) The bristles are coarse and loose c) The bristles are coarse and dense 8. Which part of the tooth is brushed? a) Front only b) Rear only c) Front and rear 9. The part of the mouth that is also brushed apart from the teeth is a) Lips b) Tongue c) Gums 10. The movement to brush your teeth facing the lips and cheeks is a) Circular motion b) Back to front movement c) Up and down movement 11. The movement to brush the surface of the teeth that smoothes food is a) Circular motion b) Back to front movement c) Up and down movement 12. How many circular motions are made: a) 6 times b) 8 times c) 10 times 13. How many times do you rinse your mouth after brushing your teeth a) One time b) Twice c) Three times 14. How much toothpaste is used to brush teeth (for elementary school children) a) As big as a green bean b) As big as corn kernels c) The size of a salak seed 15. Tooth surfaces that must be brushed are: a) Front only b) The entire surface of the tooth c) The part of the tooth used for chewing
Attitude	1. I prefer to brush my teeth when taking a shower because it is more practical 2. I don't like to brush my teeth at night before going to bed 3. I want to replace my toothbrush when the bristles are loose 4. I don't want to use toothpaste when I brush my teeth 5. In my opinion, brushing my teeth only takes 1 minute 6. I don't want to brush my teeth using the brushing technique I've been taught 7. I brush my teeth for 2 minutes 8. I brush my teeth so that my teeth don't have cavities 9. To keep my teeth from cavities I will eat less sweet and sticky foods 10. I don't need to go to the dentist every 6 months
Behavior	1. Do your teeth need to be brushed every day? 2. Do you want to brush your teeth using toothpaste that contains fluoride? 3. Have you ever used someone else's toothbrush? 4. Did you not brush your teeth last night before going to bed? 5. Did you not brush your teeth after breakfast? 6. Do you brush all surfaces of your teeth? 7. Have you brushed your teeth the right way? 8. Are you going to keep your teeth and mouth clean even without being told by your parents? 9. Do you need to brush your teeth for 2 minutes? 10. Do you need to brush your tongue after brushing your teeth?
